# Chemical control and insecticide resistance status of sand fly vectors worldwide

**DOI:** 10.1371/journal.pntd.0009586

**Published:** 2021-08-12

**Authors:** Sofia Balaska, Emmanouil Alexandros Fotakis, Alexandra Chaskopoulou, John Vontas

**Affiliations:** 1 Institute of Molecular Biology & Biotechnology, Foundation for Research & Technology Hellas, Heraklion, Greece; 2 Department of Biology, University of Crete, Heraklion, Greece; 3 Department of Crop Science, Agricultural University of Athens, Athens, Greece; 4 European Biological Control Laboratory, USDA-ARS, Thessaloniki, Greece; Institut Pasteur de Tunis, TUNISIA

## Abstract

**Background:**

Phlebotomine sand flies are prominent vectors of *Leishmania* parasites that cause leishmaniasis, which comes second to malaria in terms of parasitic causative fatalities globally. In the absence of human vaccines, sand fly chemical-based vector control is a key component of leishmaniasis control efforts.

**Methods and findings:**

We performed a literature review on the current interventions, primarily, insecticide-based used for sand fly control, as well as the global insecticide resistance (IR) status of the main sand fly vector species. Indoor insecticidal interventions, such as residual spraying and treated bed nets are the most widely deployed, while several alternative control strategies are also used in certain settings and/or are under evaluation. IR has been sporadically detected in sand flies in India and other regions, using non-standardized diagnostic bioassays. Molecular studies are limited to monitoring of known pyrethroid resistance mutations (*kdr*), which are present at high frequencies in certain regions.

**Conclusions:**

As the leishmaniasis burden remains a major problem at a global scale, evidence-based rational use of insecticidal interventions is required to meet public health demands. Standardized bioassays and molecular markers are a prerequisite for this task, albeit are lagging behind. Experiences from other disease vectors underscore the need for the implementation of appropriate IR management (IRM) programs, in the framework of integrated vector management (IVM). The implementation of alternative strategies seems context- and case-specific, with key eco-epidemiological parameters yet to be investigated. New biotechnology-based control approaches might also come into play in the near future to further reinforce sand fly/leishmaniasis control efforts.

## Author summary

Phlebotomine sand flies, mainly aggregated in the tropics and subtropics, are prominent vectors *Leishmania* parasites that cause leishmaniasis, which comes second to malaria in terms of parasitic causative fatalities globally. In the absence of human vaccines, sand fly vector control is a key component of leishmaniasis prevention and control efforts. Here, we review the current methods and active ingredients applied for sand fly chemical control, as well as the IR data (bioassay and underlying mechanisms) available for dominant sand fly vector species worldwide. Future perspectives for improving the efficiency and sustainability of sand fly control applications, in the genomics era, including the use of molecular diagnostics for evidence-based IRM and the introduction of alternative biotechnology-based approaches, are discussed.

## Introduction

Phlebotomine (Diptera: Psychodidae) sand flies represent major vectors of human and animal pathogens, such as *Leishmania* spp., *Bartonella* spp. (Carrion disease), and arboviruses, which are responsible for several diseases including sand fly fever, vesicular stomatitis, and Chandipura encephalitis [1]. Leishmaniasis, reportedly among the neglected tropical diseases with the highest global burden [2], is caused by parasitic protozoa of the genus *Leishmania* (Trypanosomatida: Trypanosomatidae), which are transmitted zoonotically and/or anthroponotically by *Phlebotomus* and *Lutzomyia* spp. in the Old and New World, respectively [1]. Three main disease forms exhibiting different clinical manifestations occur: cutaneous leishmaniasis (CL), mucocutaneous leishmaniasis, and visceral leishmaniasis (VL), also known as kala-azar, which is fatal if left untreated [3]. Leishmaniases are endemic in over 98 countries in the tropics, subtropics, and the Mediterranean basin, with approximately one million new infection cases and 20,000 to 30,000 associated deaths (mainly attributed to the VL form of the disease) occurring annually [2,3]. Over 90% of the global VL load occurs in Brazil, Eritrea, South Sudan, Sudan, Ethiopia, Kenya, Somalia, and India, whereas Brazil, Peru, Venezuela, Bolivia, Colombia, Algeria, Syria, Iran, Afghanistan, and Pakistan note the highest morbidity and mortality caused by CL [3]. Experts fear that the leishmaniasis burden may increase further in the years to come. The ongoing climate and environmental change in conjunction with the high population mobility and unplanned urbanization occurring across the globe comprise important risk factors altering the spatiotemporal distribution of vector species and their associated pathogens [4].

Early case detection and treatment are essential prerequisites for leishmaniasis control [3]. Indicatively, case management comprises the core leishmaniasis control strategy in a number of countries (i.e., Kenya, Ethiopia, and Sudan) recently witnessing a VL incidence rate increase [5,6]. However, many high-risk endemic regions lack systematic infection surveillance systems and proper diagnostic tools/expertise to conduct preventive control of the disease. Furthermore, most of the available therapeutic drugs are accompanied by severe side effects, while preventive human vaccines are still under development or clinical trial phases [7]. Hence, leishmaniasis elimination programs largely rely on vector control by means of synthetic insecticides and environmental management [8]. Notably, in many foci, sand fly control is often a by-product of antimalarial vector control efforts [8].

Environmental vector management (EVM), aiming at removing or rendering unfavorable phlebotomine sand fly potential breeding and resting microhabitats, was the standard method for controlling vector populations, until 1940. Widely implemented modifications including removing tree stumps, filling soil and indoor cracks/crevices (making them impermeable to oviposition and sand fly emergence), regular cleaning of peridomicile areas and animal shelters, and removal of organic materials and rubbish have considerably contributed to the control of vector populations [8]. EVM, depending to a large extent on local community awareness and involvement, may offer the maximum toward vector population reduction when integrated with other leishmaniasis elimination methods rather than serve as a stand-alone approach [9].

Chemical interventions, such as indoor residual spraying (IRS), space spraying (predominantly ultra-low volume (ULV) fogging), insecticide-treated nets (ITNs), topical and spatial repellents, and impregnated dog collars (IDCs), are some of the most powerful tools to control sand flies [8,10]. Nevertheless, the reliance on insecticides for controlling *Leishmania* transmission and/or other co-endemic vector-borne diseases (e.g., malaria), as well as additional pressure from agricultural insecticidal interventions, has triggered the development of insecticide resistance (IR) in key sand fly vector species’ populations in India and elsewhere [10–12], likely threatening the efficacy and operational impact of the respective control programs.

The objectives of the current article are to: (i) critically summarize sand fly chemical control interventions, including their uses, efficacy reports, and limitations; (ii) depict the global status of phlebotomine sand fly IR at the bioassay level; (iii) review the underlying IR mechanism reports across sand fly species and geographical regions; and (iv) discuss the importance and perspectives of sand fly genomics—for improving the management, efficiency, and sustainability of chemical control applications, as well as the introduction of alternative biotechnology-based vector control strategies.

## Methods

### Search methodology

We searched the published literature on sand fly chemical control, sand fly IR against major classes of insecticides, and molecular/biochemical tools for resistance tracking in wild sand fly populations. The published studies were sought from the PubMed and Web of Knowledge databases of published studies.

Key words/terms used to guide the searches included “sand flies,” “leishmaniasis,” “vector control,” “chemical control,” “insecticides,” “impregnated dog collars,” “attractive toxic sugar baits,” “insecticide resistance,” “insecticide susceptibility,” “bioassay,” “kdr mutation,” “vgsc gene,” “biochemical analysis,” and “acetylcholinesterase.” Abstracts in languages other than English (i.e., Spanish, Turkish) were translated into English and, in case of relevance, the corresponding full length manuscripts were also translated and considered for inclusion.

### Inclusion and exclusion criteria

Concerning bioassay reports, only data published as of 2000, recording mortality percentages (%) and/or lethal times (LT values) and/or lethal concentrations (LC values) of exposure to insecticides through applying WHO, CDC, or WHO/CDC modified protocols, were evaluated. Bioassay experiments on laboratory colonies, non-*Phlebotomus* or *Lutzomyia* species, and on populations for which a defined field collection date and location was not provided were excluded.

### Findings

#### Chemical control of sand flies

*Indoor residual and space spray applications*

IRS comprises the backbone of chemical control interventions in high-burden areas [13]. IRS involves coating of the inner walls and other surfaces of households and animal shelters with a residual insecticide, primarily targeting endophilic and endophagic vector species (Table 1). The intervention’s operational success relies on multifarious parameters, including commitment to the technical guidelines by properly trained spraying personnel, effective ground program supervision, the type of wall surface treated/sprayed, the quality and residual activity of the active ingredients/formulations used, the climatic conditions, and the entomological/epidemiological features of the intervention area (e.g., levels of indoor versus outdoor transmission) [14,15].

**Table 1:** Summary description of sand fly vector control practices discussed in the present review article and associated challenges/limitations.

Control strategy	MoA^a^	A.I.^b^	Targeted life stage	Operational application	Species mainly targeted/tested	Country^c^	Selected references	Challenges/Limitations
Environmental management	HM	-	Immature	Outdoors				• Breeding/immature habitats are terrestrial, widely dispersed, and not easily detected • Possible impact on local environmental features • Reliance on community’s involvement and awareness
Indoor residual spraying	LD	PY, OP, CAR (OC)	Adult	Indoors	*Lutzomyia* spp., *P*. *argentipes*, *P*. *duboscqi*, *P*. *papatasi*, *P*. *sergenti*	Bangladesh, India, Iran, Mali, Morocco, Nepal, Peru, Venezuela	[10,11,14–16,18–22]	• Protective only against intradomestic transmission (indoor-resting/indoor-biting species) • Efficacy affected by timing and rounds of spraying, wall surface type, insecticide bioavailability, etc. • Requires trained personnel and strong commitment to the technical guidelines/operational complexity
ITNs/LLINs	HC, LD	PY	Adult	Indoors	*L*. *youngi*, *L*. *ovallesi*, *P*. *argentipes*, *P*. *orientalis*, *P*. *perfiliewi*, *P*. *perniciosus*, *P*. *sergenti*, *P*. *tobbi*	Afghanistan, Bangladesh, Colombia, India, Iran, Italy, Nepal, Sudan, Syria, Turkey, Venezuela	[10,16,19,20,28–35,37–39]	• Protective only against indoor-biting species • Reliance on community’s sleeping behavior compliance • Efficacy potentially affected by vector’s biting time • High coverage required for community-wide protection effect • Limited distribution, especially in poor areas/costly
Insecticide-impregnated durable wall lining	LD	PY	Adult	Indoors	*P*. *argentipes*	Bangladesh, India, Nepal	[13,24,25]	• Costly • Installation and maintenance specialist teams required • Poor house ventilation • Large-scale evidence against sand flies required
Insecticide-treated clothing	HC (LD)	PY	Adult	Indoors/outdoors	*P*. *papatasi*		[41]	• Health risks/small amount of insecticide possibly absorbed through dermal contact
Topical/spatial repellents	HC (LD)	Natural compounds, synthetic compounds (e.g., PY, SS220, DEET, picaridin)	Adult	Indoors/outdoors	*P*. *argentipes*, *P*. *papatasi*, *P*. *perniciosus*		[10,51,52]	• Repellency effect varying between sand fly species
ATSB	LD	Neonicotinoids, spinosyns, boric acid, PY	Adult	Outdoors	*P*. *papatasi*, *P*. *sergenti*	Iran, Israel, Morocco	[46–50]	• Large-scale evidence against sand flies required • Coverage and use need to be defined • Possible effects on non-target organisms
Insecticide-impregnated dog collars	HC (LD)	PY	Adult	Outdoors	*L*. *longipalpis*, *P*. *kandelakii*, *P*. *perfiliewi*, *P*. *perniciosus*	Brazil, Iran, Italy	[10,65–67]	• High rate of coverage required to eliminate canine leishmaniasis • Regular replacement required • Costly • Collar loss often occurring
Animal feed-through insecticide baits	LD	PY, IGRs, ivermectin, fipronil	Immature/adult	Outdoors	*P*. *argentipes*, *P*. *duboscqi*, *P*. *papatasi*	India	[56,59–64]	• Field-scale evaluation required • Optimal placement of livestock shelters needed • Reliance on vector species’ host preference
Space spraying	LD	PY	Adult	Outdoors	*P*. *duboscqi*, *P*. *perfiliewi*	Greece, Kenya	[10,44,45]	• Inadequate coverage of sand fly habitats • Short residual efficacy requiring re-application • Requires trained personnel and strong commitment to the technical guidelines/operational complexity • Possible effects on non-target organisms
Insecticide-impregnated fences nets	HC (LD)	PY	Adult	Outdoors	*P*. *papatasi*, *P*. *sergenti*	Israel	[42,43]	• Large-scale evidence against sand flies required
Biological/chemical larvicides	LD	Microbial larvicides (*Bt*), IGRs, OPs	Immature	Outdoors	*L*. *longipalpis*, *P*. *argentipes*, *P*. *duboscqi*, *P*. *martini*, *P*. *papatasi*	Argentina, Bangladesh, Kenya	[10,19,54,55]	• Breeding/immature habitats are terrestrial, widely dispersed and not easily detected

^a^MoA, Mode of Action; HM, habitat modification/ manipulation; LD, reduction of sand fly longevity and population density; HC, reduction of host–sand fly contact.

^b^A.I., Active Ingredient; PY, pyrethroid; OP, organophosphate; CAR, carbamate; OC, organochlorine; *Bt*, *Bacillus thuringiensis*; IGR, insect growth regulator.

^c^The list refers to countries where field trials of the respective control strategies have been conducted.

Dichlorodiphenyltrichloroethane (DDT)-based IRS, often integrated within malaria elimination programs, was the mainstay of sand fly control since 1944 and for several decades to follow [8], largely contributing to leishmaniasis transmission suppression in this period in countries such as India, Nepal, Iran, Syria, Italy, Greece, and Peru [10,11,16]. However, the introduction of restrictive measures against several organochlorines (OCs) in the 1970s, due to their high toxicity risk (for human and animal health) and environmental persistence, in conjunction with reports on DDT resistance in sand fly vector populations in disease-endemic regions (e.g., India) [11], imposed a shift toward the use of safer insecticides. To date, pyrethroids are the primary insecticide class used in IRS applications around the globe [17]. Indicatively, interior wall spraying with lambda-cyhalothrin in villages of the Peruvian Andes [10] and deltamethrin in regions of the Indian subcontinent [14,18] resulted in a 70% or higher household-level reduction in the abundance of *Lutzomyia* spp. and *Phlebotomus argentipes* sand flies, respectively, for up to 6 months post-intervention. During a controlled trial in villages of Bangladesh, 2 rounds of alpha-cypermethrin spraying resulted in an average maximum of 75% *P*. *argentipes* population density reduction (14 months post-first round and 2 months post-second round) [19]. A markedly lower vector density was reported in pyrethroid-treated kala-azar–endemic districts in eastern Nepal (mean drop of 94.5% in the number of sand flies trapped per night/per house over a 5-month period post-intervention) [9]. Furthermore, alpha-cypermethrin spraying in areas of northern Morocco significantly limited CL cases from 9 to 0 per 1,000 inhabitants [15], yet no considerable effect was observed on the abundance of *Phlebotomus sergenti*, the main CL vector in the region.

Several field studies have reported suboptimal results upon IRS operations with the duration of insecticides’ residual efficacy being the critical point. For example, recent small-scale pyrethroid IRS trials targeting *P*. *argentipes* in high-risk areas of the Indian subcontinent failed to sustain a significant population reduction for more than 12 weeks [14,20]. Similar results were reported following lambda-cyhalothrin IRS applications against *Lutzomyia* vectors in Margarita Island, Venezuela [21,22].

Insecticide-treated durable wall lining (DWL) is an alternative type of indoor residual intervention, relying on the application of an insecticide-treated thin polyethene net covering partially or completely the inner wall surfaces [23] (Table 1). This new technology was created to improve the residual effect of insecticides commonly observed in IRS treatments and for the practicality of the application. Although we lack epidemiological data showing the method’s impact on leishmaniasis focal incidence, recent comparative analyses of different interventions (DWL, IRS, and slow-release insecticide tablet impregnation of bed nets) in southern Asian countries evaluated DWL as the most powerful tool for reducing the abundance of local *P*. *argentipes* populations [13,24]. Indicatively, Huda and colleagues [25] recorded a 63% to 73% decrease of the indoor female sand fly density in the DWL intervention clusters 1 month post-installation, while the material retained its insecticidal activity for at least 12 months. Overall, linings have been accepted in a number of traditional settings (particularly in isolated villages), where IRS logistics pose a significant challenge. However, the intervention’s high cost along with the deposition and handling of the large volume of insecticide-treated plastic surfaces following their use remain critical issues possibly having triggered the cessation of the manufacturing processes.

Indoor space spraying (ISS), targeting resting or flying individuals, has been performed against malaria mosquitoes especially as an emergence response, offering a one-off knockdown (KD) effect (often also exploited for monitoring purposes), followed by a short residual effect. Even though ISS adulticidal outcome on mosquito vectors has been assessed [26], there is currently no evidence available of its potential operational efficacy against sand fly vectors.

*Insecticide-treated bed nets*

ITNs and long-lasting ITNs (LLINs) comprise important intradomestic *Leishmania* transmission control tools through acting as a toxic physical barrier against blood-seeking sand flies [27], offering both personal- and community-level protection upon high net coverage/distribution (Table 1). Their field efficacy has been evidenced in countries of the Mediterranean basin, Latin America, and Africa [10]. In community-wide trials conducted in Sudan and countries of the Middle East, broad distribution of ITNs reduced the leishmaniasis burden by 59% to 98% for at least 1 year [28–30]. Specifically in Iran, epidemiological evidence revealed a close to elimination of CL incidence rate in high-risk regions of the country where pyrethroid-treated bed nets and curtains had been in use for 1- or 2-year trials [16,30–32]. A remarkable drop in the annual CL incidence (compared to control regions) was also reported upon ITNs deployment in 2 intervention areas in Sanliurfa, Turkey [33]. Mondal and colleagues [34] and Chowdhury and colleagues [35] reported a 66.5% and 46.8% VL incidence reduction in rural areas of Bangladesh after 1 and 3 years of ITNs application, respectively.

A small number of community-scale ITNs/LLINs trials, alongside the recorded reduction of leishmaniasis incidence, also report a reduction of the local vector populations’ abundancy [9,20,30,36]. Indicatively, Chowdhury and colleagues [19] observed a maximum drop of 78% in *P*. *argentipes* household density following village trials in Bangladesh. A similar effect (60% reduction) against the same vector species, lasting for at least 18 months, was observed by Mondal and colleagues [34], accompanying deltamethrin impregnation of the existing bed nets. In addition, trials conducted in the Indian subcontinent have shown a sand fly density drop persisting for 9 months in the ITN clusters [24].

The cotreatment of LLINs with insecticide synergists, such as piperonyl butoxide (PBO; a known inhibitor of P450 monoxygenases), appears a highly promising supplement for vector control in vector-borne disease (VBD) endemic areas of intense pyrethroid resistance. Gunay and colleagues [37], having assessed the protective efficacy of Olyset Plus LLIN (2% permethrin and 1% PBO) in a hyperendemic village in Cukurova region, Turkey, reported a 92% protection rate from *Phlebotomus tobbi* bites and a decrease of CL prevalence up to 4.78%, during the post-intervention year.

In some cases, village-wide distribution of LLINs in India and Nepal did not significantly impact the infection rates nor affected the transmission occurring in these regions’ ecological settings [38]. Operational issues, short residual efficacy, lack of community compliance, and/or possible IR/tolerance, including potential behavioral shifts in vector species’ feeding patterns, are likely to be responsible for such unsatisfactory results [13,20,38,39]. Indicatively, the KALANET project, a cluster randomized controlled trial of mass ITNs distribution implemented in India and Nepal, failed at remarkably diminishing vector’s survival and, thus, VL infection rates, due to *P*. *argentipes* exhibiting a more intensely zoophilic and exophagic behavior than previously observed in some endemic biotopes [39,40].

*Other insecticide-treated materials (ITMs) for personal and community protection*

In VBD high risk–prone regions, workers, the public, and the military commonly utilize clothing impregnated with insecticides [41] (Table 1). Permethrin is the preferred active ingredient (for treating clothing), due to its low health risk, combined with its insecticidal and repellent effect. Although the protective efficiency of insecticide-treated clothing is substantial against mosquitoes, we lack robust evidence concerning sand flies. The few available studies report a leishmaniasis protection rate varying from 0% to 79% [41].

In Jordan Valley, Iran, field trials with pyrethroid-impregnated vertical fine mesh nets serving as physical barriers against sand flies entering inhabited areas [42,43] reduced the abundance of sand flies trapped in the enclosed areas by over 60% compared to pre-fence net placement. These preliminary results suggest that this could be a valuable additive measure integrated in comprehensive chemical control campaigns.

*Outdoor residual and space spray applications*

Insecticide spraying on trees and vegetation around human dwellings, animal shelters, or in barrier zones targeting flying, breeding, or resting insects is not a widely applicable measure against *Leishmania* vectors, as it comes with important limitations due to inadequate habitat coverage and poor insecticide residual activity [10] (Table 1).

However, multiple trials of cold fogging or ULV space spray insecticide applications in countries of Latin America have demonstrated satisfying results in the reduction of sand fly population and leishmaniasis incidence within the intervention sites [10]. In a recent study carried out in western Kenya, malathion and synthetic pyrethroid + PBO formulations tested in ULV applications suppressed the local sand fly populations (mainly, *Phlebotomus duboscqi*) by at least 50%, immediately after spraying [44]. Encouraging results were also published by Chaskopoulou and colleagues [45], where *Phlebotomus perfiliewi* populations decreased by 66% in heavily infested animal facilities of Greece, 24 hours post-high rate ULV ground application of a deltamethrin-based formulation.

*Attractive toxic sugar baits (ATSBs)*

Composed of a sugar source, an attractant and an oral toxin/insecticide, attractive toxic sugar baits (ATSBs) are applied against indoor or outdoor insect vector populations, targeting their sugar-seeking behavior (Table 1). This method appears to act on both male and female insects during their adult life span, by killing them either directly or through dissemination [46]. ATSBs have been applied in small-scale field trials in multiple formats: (i) sprayed on vegetation; (ii) coating barrier fences around villages; and (iii) incorporated into bait stations, overall generating encouraging preliminary results. In zoonotic CL-endemic regions of the Middle East, specifically Iran and Israel, vegetation and barrier fencing ATSB applications including 1.0% w/w boric acid led to a close to 90% reduction of the *Phlebotomus papatasi* populations [47–49]. In addition, evaluation of the residual activity of ATSB-treated barrier fence nets in central Iran revealed their potency for at least 60 days post-installation, causing at that time point a mortality rate of 51% in the field-caught, cone bioassay–exposed *P*. *papatasi* sand flies [50]. Finally, Qualls and colleagues [46], following a 4-week period of ATSB vegetation spraying and bait station deployment in a Moroccan agricultural area, recorded an 83% decrease in *P*. *papatasi* and *P*. *sergenti* populations within the treated sites, with negligible impact on non-target insects.

*Topical and spatial repellents*

Natural or synthetic compounds with repellent activity are commonly applied against several insect vectors either at the household level or on skin/clothing for personal protection, hindering the vector–human interaction (Table 1). Spatial repellents appear in different formats, such as candles, coils, and sprays.

Laboratory pilot trials on the repellency effect of several chemical compounds, such as picaridin, SS220, N,N-diethyl-3-methylbenzamide (DEET), and ethyl-butylacetylaminoproprionate (IR3535), proved that they could serve as biting deterrents against different sand fly species (e.g., *P*. *papatasi*, *P*. *duboscqi*, *Phlebotomus perniciosus*). However, the repellency effect and duration seem to vary depending on the targeted species [51]. Active natural ingredients derived from plants, such as *Ricinus communis*, *Solanum jasminoides*, *Capparis spinosa*, and *Geranium* spp., have also been tested against sand fly laboratory colonies, displaying repellent and/or insecticidal properties [52], yet the potential effect of natural ingredients’ field application against local sand fly population abundance and/or protection from sand fly bites remains greatly understudied.

*Applications targeting sand fly immature stages*

The control of sand flies at their immature stages mainly relies on habitat modification and manipulation (within the EVM context). Sand flies breed in a wide variety of terrestrial sites that are not easily detectable nor well characterized, making the use of larvicides difficult (Table 1). However, an increasing effort to develop tools for easy identification of sand fly larval habitats [53] may greatly enhance the precision and efficacy of larviciding interventions.

Within the framework of larval chemical control, Gómez-Bravo and colleagues [54] evaluated the Dragon Max formulation (i.e., a combination of 2 active ingredients: permethrin + pyriproxyfen (insect growth regulator (IGR)) versus a permethrin (only) formulation, in field applications in Clorinda, Argentina. Chicken coops and surrounding vegetation were sprayed, covering potential phlebotomine breeding sites. The denoted drastic decrease in the abundance of *Lutzomyia longipalpis* exposed to Dragon Max lasted for 21 weeks and was not observed in areas exposed to permethrin-only treatments. In another study conducted in Bangladesh, no significant reduction was observed following chlorpyrifos (organophosphate (OP)) spraying in sand fly oviposition sites, i.e., shady, moisture, and rich in organic materials (e.g., cow dung) places, outside human dwellings, and cattle shades [19].

Other active ingredients of biological origin, such as formulations of *Bacillus thuringiensis* var. *israelensis* (*Bti*) and *Bacillus sphaericus (Bsph)* have been also tested against sandflies. *Bti* has been shown to cause significant mortality in both larval and adult stages, under laboratory conditions, whereas a similar effect was noticed upon *Bsph* field spraying on vegetation [10]. Application of the entomopathogenic fungi *Metarhizium anisopliae* in termite mounds used for resting/breeding sites led to 3- to 10-fold increase in mortalities of the sand fly vectors *Phlebotomus martini* and *P*. *duboscqi*, 9 weeks post-application in Rabai, Kenya, while a reduced adult sand fly longevity was also observed [55]. However, the operational efficiency of such interventions is yet to be evidenced.

*Insecticide zooprophylaxis and animal protection*

Systemic treatment of livestock (e.g., cattles) and/or other sand fly vertebrate reservoir hosts with orally administered insecticides displays promising potential in facilitating the control of outdoor blood-feeding adults and/or feces-feeding larvae (Table 1). To date, several laboratory studies have revealed reduced survival of *Phlebotomus* spp. larvae upon feeding on *Mesocricetus auratus* hamsters’ feces treated with IGR larvicides, such as diflubenzuron, novaluron, and juvenile hormone analogues [56]. Ivermectin, widely used against zoophilic and anthropophilic/anthropozoophilic insect vectors and the pathogens they transmit [57,58], has proved a powerful rodent feed through insecticide in *Leishmania* vector control laboratory trials [59]. Additionally, the significant activity of the systematic use of ivermectin-treated livestock against *Anopheles* mosquito vectors’ survival in field conditions may have a similar impact on sand fly populations [60]. Similarly, fipronil single dose–treated rodent (*Meriones shawi*, *Rattus rattus*, *Bandicota bengalensis*, etc.) and cattle (*Bos taurus*, *Bos indicus*) baits have been evaluated, under both laboratory and field conditions, against *Phlebotomus* spp. *(*i.e., *P*. *argentipes*, *P*. *papatasi)*, causing an effect of almost total larvae/adult mortality (80% to 100%) lasting for 3 to 6 weeks [61–64]. Protection of dogs, the principal *Leishmania infantum* reservoir host, from sand fly bites is commonly mediated by slow-release insecticide IDCs that maintain an antifeeding and insect-killing activity for approximately 6 to 8 months (Table 1). So far, collars impregnated with deltamethrin and flumethrin/imidacloprid have been developed and validated both in laboratory and field studies [65]. Recently, Yimam and colleagues [66] systematically reviewed the effectiveness of IDCs. Mass use of IDCs has efficiently reduced the risk of canine leishmaniasis (canL) transmission by 46% to 86% during intervention trials in Italy, Iran, and Brazil. High collar coverage (approximately 90%) within the target area was shown to be essential to abate canL occurrence. However, this comes at a considerable economic cost and is not always feasible [66]. Interestingly, community-wide deployment of deltamethrin IDCs in Iran demonstrated an additional protective efficiency against infantile *L*. *infantum* infections [67].

Biocidal spot-on formulations such as lotions and sprays comprise complementary prophylactic measures mostly applied at an individual–animal level [68].

### Insecticide resistance—Bioassay data

Historical data on IR phenotypes of *Lutzomyia* and *Phlebotomus* vector species have been reviewed by Alexander and Maroli [10], Dhiman and Yadav [11], and Rocha and colleagues [12]. Here, we summarize global reports since 2000 and the most recent bioassay data (2016 to 2020) from southeastern Asia.

Despite the prolonged exposure of sand flies to insecticides [17], our knowledge on their susceptibility status remains narrow. Notably, the available bioassay data are geographically aggregated in the highest leishmaniasis burden countries with important spatiotemporal data coverage gaps in all continents. To date, Bihar and West Bengal, the main epicenters of VL transmission in India, are the only regions with systematic data coverage over the years [11,12]. Limitations in data availability can partially be attributed to challenges in sand fly collection and rearing and to the lack of sand fly standardized bioassay protocols (i.e., established diagnostic doses and exposure times) [12,69]. Hitherto, the majority of susceptibility tests have been carried out following WHO tube or CDC bottle protocol and the corresponding diagnostic doses derived from mosquitoes [12,70,71], while very few studies have implemented WHO or CDC bottle bioassay protocols for establishing lethal concentrations and exposure times specific and up to date against sand flies [69,72–74]. Overall, the straight comparison and consistent interpretation of sand fly bioassay results generated in different studies are difficult to be made, due to (i) the remarkable variation in the bioassay protocols applied; and (ii) the lack of susceptible laboratory colonies tested simultaneously with the field-caught populations (in the vast majority of studies) [12].

**Fig 1:**
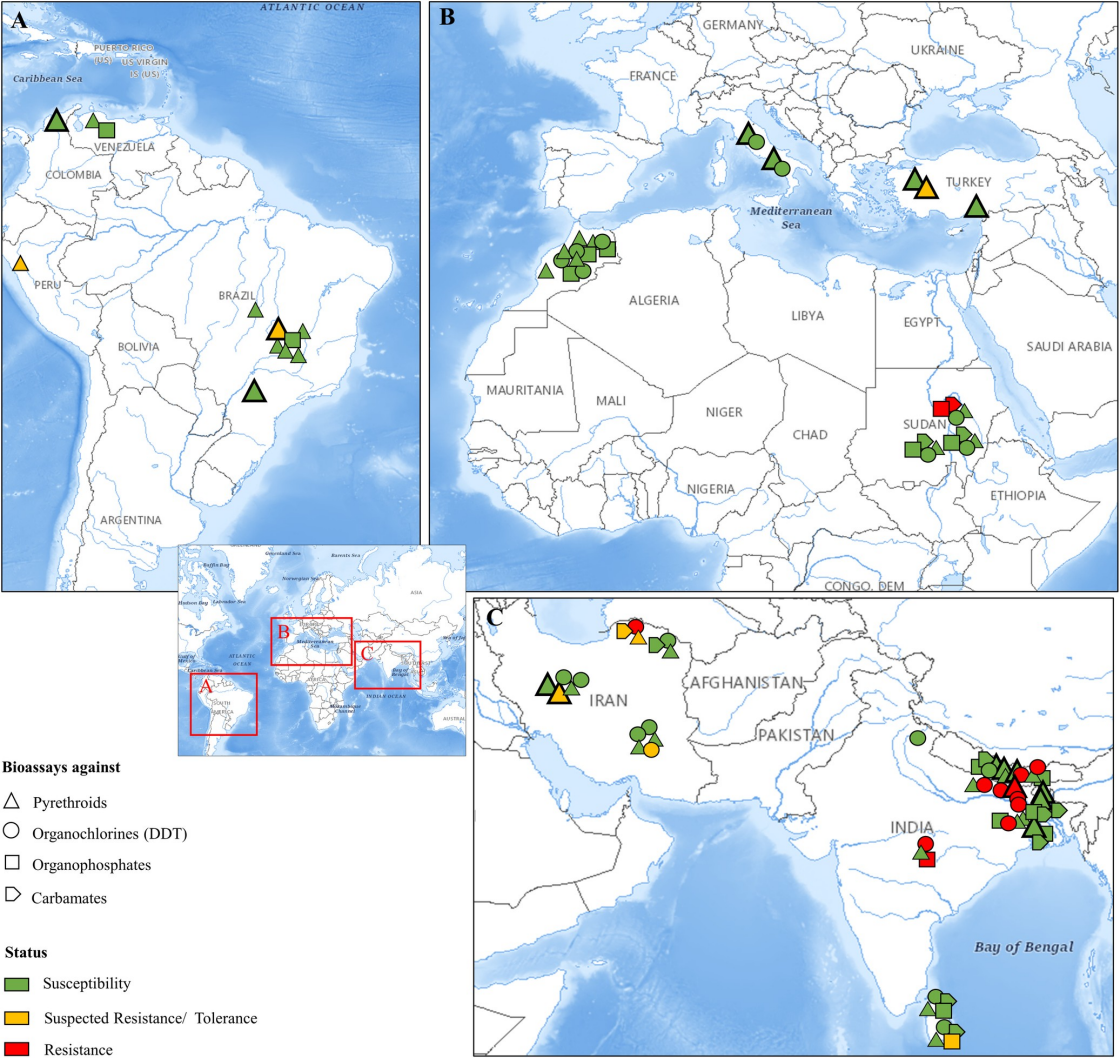
Global geographical distribution of IR bioassay reports in *Lutzomyia* and *Phlebotomus* sand fly vector species. The global map focuses in regions with data coverage since 2000: (A) Latin America, (B) Mediterranean basin and north-central Africa, and (C) the Middle East and southeastern Asia. The sand fly species analyzed per region are (A) *L*. *longipalpis*, *L*. *evansi*, and *L*. *peruensis*, (B) mainly, *P*. *papatasi*, *P*. *sergenti*, and *P*. *tobbi*, and (C) mainly, *P*. *papatasi*, *P*. *sergenti*, and *P*. *argentipes*. Data correspond to WHO tube or CDC bottle bioassays in adult field-caught sand fly populations, against compounds of the 4 main insecticide classes. For WHO and CDC bioassay experiments using insecticide discriminating doses, the resistance status is determined by the mortality percentage (%) recorded 24 hours post-exposure (1 hour of exposure), as follows: ≥98% shows susceptibility (green), 90%–97% indicates the possibility of resistance (yellow), and <90% denotes resistance (red). Regarding the cases where (i) KD rates (%) at 1 hour of exposure are given; (ii) other than discriminating doses were tested; or (iii) dose–or time–response KD/mortality curves were provided, the susceptibility/resistance status is presented as defined by the respective authors. The symbol size varies depending on the number of insecticides of the same class tested against a specific population. In cases of differences in the population’s response against these insecticides, the less sensitive condition is presented. A detailed dataset for each bioassay experiment is provided in S1 Table. Maps were obtained from USGS (https://apps.nationalmap.gov/viewer/). DDT, dichloro-diphenyltrichloroethane; IR, insecticide resistance; USGS, US Geological Survey.

Throughout the text and in **Fig 1**, population data extracted from WHO tube or CDC bottle bioassays are classified as susceptible, suspected resistance/tolerance, and resistance based on the percentage (%) of mortality recorded 24 hours post-insecticide exposure (i.e., 60 minutes of exposure at the putative diagnostic doses extrapolated from mosquitoes [70,71]). As described in WHO/CDC guidelines, mortality ≥98% shows susceptibility, 90% to 97% indicates the possibility of resistance, and <90% denotes resistance. In all other cases where (i) non-discriminating doses were tested; or (ii) other than mortality percentage metrics were provided, the susceptibility/resistance profiles of the corresponding populations are presented as defined by the respective authors. The bioassay records included in the review are presented per region, and all respective assay details (including sand fly species, bioassay protocols deployed, and generated test values) are synopsized in **S1 Table**.

#### Indian subcontinent

In India, important leishmaniasis vector species appear resistant to a number of public health insecticides. The most alarming problem is that of DDT resistance, documented in *P*. *argentipes* and *P*. *papatasi* populations from West Bengal and Bihar. Indicatively, a series of studies report DDT 4% exposure mortality rates ranging from 43% to 100% [11,75–77] and KDT_50_ (i.e., time of exposure causing 50% KD of the tested population) values exceeding 30 minutes [76] in both vector species. Resistance has also been recorded against the OC dieldrin in West Bengal and Uttar Pradesh *P*. *papatasi* populations [10–12]. The only recent record (from 2000 onward) of pyrethroid resistance in the country was documented in Bihar *P*. *argentipes* populations, with mortality rates of 56% and 84% against deltamethrin and alpha-cypermethrin 0.05%, respectively [75]. Both *P*. *argentipes* and *P*. *papatasi* Indian populations have occasionally displayed evidence of resistance against carbamates (i.e., propoxur) and OPs (i.e., malathion) [11], yet the majority of bioassay records against these compounds denoted susceptibility [11,76].

Elsewhere in the Indian subcontinent, evidence of DDT resistance has been recorded in *P*. *argentipes* specimens from Sunsari district, Nepal (with a DDT 4% KD value of 51% and mortality 62%), whereas the same population appeared sensitive to deltamethrin 0.05% [11]. In 2015 to 2016, Chowdhury and colleagues [78] evaluated the sensitivity status of *P*. *argentipes* in VL-endemic areas of southeastern Nepal and Bangladesh revealing KD percentages >81% and 100% mortality following exposure to alpha-cypermethrin 0.05%, deltamethrin 0.05%, lambda-cyhalothrin 0.05%, permethrin 0.75%, malathion 5%, and bendiocarb 0.10%.

The sole study depicting (through WHO dose response assays) the phenotypic status of *P*. *argentipes* populations from Sri Lanka showed DDT, malathion, propoxur, and deltamethrin sensitivity in 3 of the 4 tested populations, with LC_100_ (i.e., insecticide concentration leading to 100% mortality of the tested population) values of 0.8%, 0.9%, 0.017%, and 0.007%, respectively [79]. The Mamadala (fourth) population displayed lower sensitivity against malathion (LC_100_ 2%), DDT (LC_100_ 1.5%), and propoxur (LC_100_ 0.03%), possibly indicating incipient resistance associated with the prolonged exposure of the district population to OPs and carbamates for public health and agricultural purposes.

#### Mediterranean basin and the Middle East

Recent studies have evaluated the susceptibility status of vector populations in Italy, Turkey, Morocco, Sudan, and Iran. In 2002, Maroli and colleagues [80] reported the susceptibility of recently colonized *P*. *papatasi* and *P*. *perniciosus* populations from Italy against DDT (2%), lambda-cyhalothrin (0.06%), and permethrin (0.2%). In Turkey, 2 studies from Karakus and colleagues, in 2016 and 2017 [81,82], evaluated the response of mixed field-caught *Phlebotomus* and *Sergentomyia* populations from leishmaniasis-endemic villages of Mugla, Aydin, and Adana Provinces against deltamethrin 0.05% and permethrin 0.75%. The dose–response WHO bioassays showed sensitivity to both compounds for the Adana [81] and Aydin [82] populations, the latter unexposed to any prior insecticidal pressure displaying KDT_50_s values <23 minutes and 100% mortality. On the contrary, in Mugla, where pyrethroid-based mosquito control programs have been in operation for a long time, the field population proved tolerant to both deltamethrin and permethrin with KDT_50_ values <37.5 minutes and mortality rates below 93.3% [82].

Faraj and colleagues [83] assessed the insecticide susceptibility status of *P*. *papatasi* and *P*. *sergenti* populations from CL-endemic villages of Morocco through applying the standard WHO protocol and discriminating doses established for sand flies (1981) [84]. KD rates of 100% (at the 1-hour exposure time point) against DDT (4%), lambda-cyhalothrin (0.05%), and malathion (5%) were recorded, denoting susceptibility of both vector species to these compounds. Although within the susceptible status frame, the tested populations exhibited noticeable differences in their KDT_50_ values (ranging from 11 to 33.8 minutes) with the higher values reported in populations from areas exposed to local DDT and pyrethroid-based malaria/leishmaniasis control spraying operations [83].

Hassan and colleagues [85] analyzed the resistance status of 3 *P*. *papatasi* populations from Sudan against compounds of all 4 major insecticide classes. The White Nile area and Rahad Game Reserve Camp populations showed full sensitivity against all tested compounds, while the population from Khartoum (an area with intense malaria vector control programs) displayed resistance against malathion (5%) and propoxur (0.10%) (KDT_95s_ > 24 hours and mortality rates < 20%) and suspected tolerance against DDT (4%) and permethrin (0.75%) (KDT_95_ values of 85 minutes and 194 minutes, respectively).

In Iran, *P*. *papatasi* and *P*. *sergenti* populations collected in 2010 from Bam district, Kerman Province, showed susceptibility against DDT 4% and deltamethrin 0.05% [86]. Likewise, *P*. *papatasi* populations from Badrood district (a leishmaniasis-hyperendemic focus of Esfahan Province) sampled in the same year displayed susceptibility to cyfluthrin (0.15%), permethrin (0.75%), deltamethrin (0.05%), lambda-cyhalothrin (0.05%), and DDT (4%) (mortalities >98%) [87,88]. However, a follow-up study in Badrood district in 2015 reported *P*. *papatasi* incipient resistance (mortalities <98%) against DDT, permethrin, and deltamethrin [89].

Signs of tolerance were also reported in *P*. *sergenti* populations from North Khorasan villages (sampled in 2015) against DDT (4%), permethrin (0.75%), and bendiocarb (0.10%) [90]. Overall, despite the restriction of DDT usage in Iran over the last decade, it appears that both *P*. *papatasi* and *P*. *sergenti* populations retain greater sensitivity to compounds of other insecticide classes (i.e., pyrethroids and carbamates) compared to DDT.

#### South America

The pyrethroid and OP insecticide susceptibility profiles of several *Lutzomyia* species have been evaluated in South America, where insecticide compounds of these classes are the most commonly deployed for IRS in the continent [17]. *L*. *longipalpis* populations from Monte Claros, Brazil, showed resistance to deltamethrin 0.05% (63.7% mortality) and possible permethrin tolerance, as increased LT_50_ (time of exposure causing 50% mortality of the tested population) permethrin (0.10%) values were recorded compared to other field populations tested (Lapinha Cave) [91]. In 2015, Rocha and colleagues [92] performed dose–response CDC bottle bioassays against alpha-cypermethrin in mixed *Lutzomyia* spp. populations (*L*. *longipalpis* was the most frequent species) collected from Minas Gerais localities, Brazil. All populations were characterized susceptible (with LC_50_ values ranging from 1.48 to 2.57 μg/mL), despite the systematic appliance of alpha-cypermethrin in local sand fly control programs. Recently, González and colleagues [74], having deployed a modified WHO tube assay, reported susceptibility (100% mortality and KDT_50_ values <31 minutes) of a Sao Paulo, Brazil, *L*. *longipalpis* population against deltamethrin 0.50% and lambda-cyhalothrin 0.05%. Last but not least, a number of WHO/CDC dose–response bioassay studies have been conducted in Brazil, Peru, Venezuela, and Colombia, in order to define the LC values (LC_50_, LC_95_, or LC_99_) for *L*. *longipalpis* [93], *Lutzomyia evansi* [94,95], and *Lutzomyia peruensis* [96] field populations against commonly used insecticides. The respective test values are given in **S1 Table**.

### Mechanisms of insecticide resistance

The underlying mechanisms of resistance in sand flies remain largely unknown. Most molecular studies focus on target-site mutations with fewer reports on metabolic resistance. Cuticular or behavioral resistance mechanisms, which have been reported and described in other insect vectors [97,98], have not been investigated in sand flies. Nevertheless, the gradual shift of *P*. *argentipes* to a more exophagic–zoophagic pattern (i.e., cattles shades) in Indian endemic foci, such as West Bengal, is speculatively attributed to the long-term indoor DDT exposure, consisting a behavioral adaptation hint [39].

#### Target-site resistance

**Fig 2:**
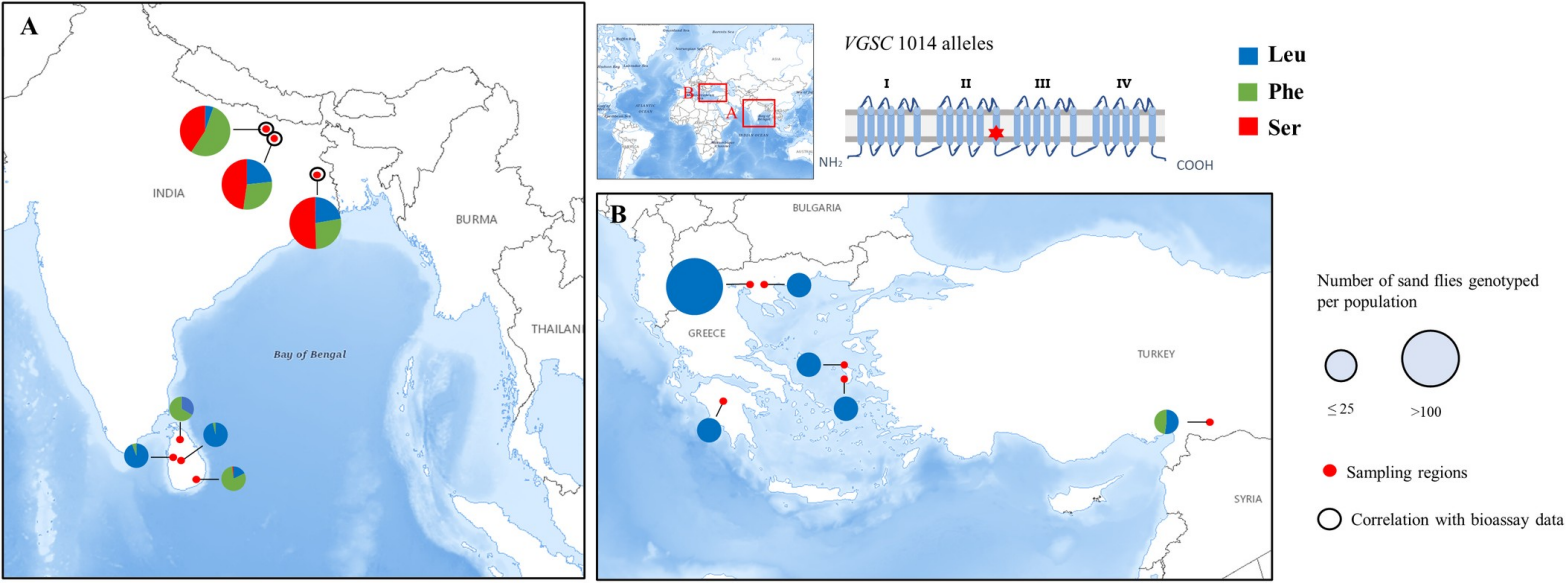
Global geographical distribution and respective allelic frequencies (%) of the *kdr* mutations at *VGSC* locus 1014 in sand fly populations. Molecular analyses refer to (A) *P*. *argentipes* populations from India and Sri Lanka and (B) mixed *Phlebotomus* spp. populations from Greece and *P*. *papatasi* populations from Turkey. The size of the pie charts is proportional to the number of specimens genotyped per population. Red dots denote the sampling regions in the 4 countries. Genotyping data correlated with results of bioassay experiments are marked with a black circle. The allelic frequencies, the number of total specimens analyzed per population, and the species included are given in detail in S2 Table. Maps were obtained from USGS (https://apps.nationalmap.gov/viewer/). *kdr*, knockdown resistance; Leu, leucine (wild-type allele); Phe, phenylalanine (mutant allele); Ser, serine (mutant allele); USGS, US Geological Survey; *VGSC*, voltage-gated sodium channel.

Resistance to DDT and pyrethroids (type I and II) has been correlated with multiple point mutations in the voltage-gated sodium channel (*VGSC*) gene (i.e., knockdown resistance (*kdr*) mutations), which are reportedly widespread and well conserved in several insects of public health and agricultural importance [99]. Among them, 2 polymorphisms at the *VGSC* transmembrane segment IIS6 locus 1014, L1014S (from TTA to TCA), and L1014F (from TTA or TTG to TTT or TTC), both functionally associated with reduced sensitivity against type I and type II pyrethroids [100], have been found in phlebotomine sand flies **(Fig 2 and S2 Table)**. The available *kdr* mutation reports amount to: (i) the presence of both 1014F and 1014S mutations in the major VL vector *P*. *argentipes* in Bihar and West Bengal states, India, with allelic frequencies ranging from 27% to 54% [75,76]; (ii) 1014F occurrence, at a close to 50% allelic frequency in a small number of *P*. *papatasi* specimens from Sanliurfa, an important CL focus in Turkey [101]; and (iii) the presence of the 1014F and 1014S mutant alleles in *P*. *argentipes* specimens from Sri Lanka at overall frequencies of 53.77% and 1.88%, respectively [79].

Sequencing analyses for the presence of other known *kdr* mutations (i.e., *VGSC* I1011M, V1016G, and F1020S) in sand fly populations from India, Greece, and Turkey reported their absence (in the respective specimens) [75,76,79,102], including the non-detection of 1014F/S mutations in the Greek populations (comprising of *Phlebotomus neglectus*, *P*. *perfiliewi*, *Phlebotomus simici*, and *P*. *tobbi* specimens) [101,102].

Gomes and colleagues [75] and Sardar and colleagues [76], investigating sand fly resistance in important foci of VL in India, correlated the recorded DDT and, possibly, pyrethroid type II resistance phenotypes with the *kdr* mutation 1014F in homozygosity and to a lesser extent with 1014S. Notably, in India, Sri Lanka, and Turkey (where the *kdr* mutations have been recorded), DDT and pyrethroids have been intensely used against mosquitoes or other disease vectors [11,17,81], possibly imposing strong selection pressure on the local sand fly populations, resulting in the reported 1014F/S allele frequencies.

OP and carbamate resistance has been correlated (in several insects) with reduced acetylcholinesterase (AChE) sensitivity attributed to specific point mutations in the *ace1*,*2* genes [99]. Sand fly biochemical studies have revealed possible reduced AChE susceptibility in *P*. *argentipes* field populations from Sri Lanka [79,103] and AChE insensitivity (>80% residual activity) against malathion and propoxur in a *P*. *papatasi* population from Khartoum, Sudan [85]. The presence of known *ace* mutations (e.g., G119S, F455W), conferring target-site insensitivity to OPs and carbamates in mosquitoes and other insects [104], is yet to be examined in sand flies. However, taking into account: (i) the conserved (among a number of dipterans) *ace* gene motifs in the *L*. *longipalpis* AchE gene [105]; (ii) the *L*. *longipalpis* and *P*. *papatasi* AChEs’ common amino acid identity (>85%) with those of major mosquito vectors (e.g., *Culex pipiens*, *Aedes aegypti*) [105,106]; and (iii) the similar *P*. *papatasi* AChE biochemical properties to those of *Ae*. *aegypti* recombinant AChE [106], it is possible that such mutations may also occur in wild sand fly populations.

In contrast to mosquitoes and agricultural pests, target-site resistance mutations against neonicotinoids, IGRs, or *Bt* active ingredients have not been explored in sand flies.

#### Metabolic resistance

Metabolic detoxification, which inactivates and sequesters insecticides [99,107], is a common mechanism of IR (found across multiple insects), which can cause operationally relevant resistance levels, especially when combined with target-site resistance mutations [108]. Fawaz and colleagues [109] highlighted the role of metabolic detoxification pathways in a permethrin-resistant *P*. *papatasi* colony (established under laboratory selection); biochemical and molecular analyses revealed that the observed resistance was mediated by elevated oxidase and esterase activity levels.

Quantitative changes of esterase activity levels were also reported in OP- and carbamate-resistant *P*. *argentipes* populations originating from Delft Island, Sri Lanka [103]. Last but not least, glutathione-S transferase and esterase activity levels above the discriminating values given for mosquitoes were recorded in *P*. *argentipes* populations sampled across Sri Lanka [79], although the relevance of this dataset with resistance remains to be validated.

### Outlook and future perspectives

Leishmaniasis prevention/control campaigns are required and implemented in multifaceted risk-prone settings displaying a wide diversity of entomological, epidemiological, ecological, and socioeconomic factors. Several control tools including IRS, ITNs, and other ITMs, as well as outdoor insecticidal interventions are currently used for sand fly control. Nevertheless, no intervention implemented under a “stand-alone–silver bullet” approach may suffice to sustainably prevent leishmaniasis transmission. Uptake of the IVM concept, supporting the case-specific deployment of control measures (alone or in combination), tailored to the local entomological, ecological, and epidemiological features of each endemic focus, under an evidence-based, cost-effective, sustainable, and ecologically friendly approach, may greatly enhance the performance of sand fly control programs. However, critical evaluation of control methods—including quality of trial designs—is lacking. Within this framework, the establishment of guidelines for leishmaniasis vector control supporting the design and assessment of field trial control interventions may greatly facilitate the realization of high-quality IVM programs.

The standardization of sand fly–specific WHO and CDC bioassay protocols and the establishment of operationally relevant diagnostic doses against the active ingredients used for their control are essential for evidence-based management of chemical control interventions [12]. In addition, the identification of robust molecular markers associated with IR, as well as additional population traits (e.g., species composition and presence of *Leishmania* parasites), and the subsequent development and application of individual or multiplex/integrated molecular diagnostic tools are prerequisites for systematic entomological monitoring and evidence-based control.

The arsenal of sand fly control interventions may be reinforced in the near future as alternative biotechnology-based control methods might be developed or adapted from other vectors. The ongoing effort to sequence the genome of several sand fly species should stimulate research, allowing the devise of new strategies. For example, gene editing via CRISPR/Cas9 methodology in *P*. *papatasi* and *L*. *longipalpis*, aiming to target leishmanial vector competence, has been recently attempted [110]. Furthermore, *Phlebotomus* and *Lutzomyia* species have been found naturally infected with *Wolbachia* strains [111], and a potential for the use of *Wolbachia*, alone or in combination with sterile insect technique, has been indicated [112]. Gut microbiome alterations have also been shown to impair vectorial capacity in sand flies [113]. Nevertheless, the fact that more than one sand fly species may be prevalent in leishmaniasis-endemic regions might limit the potential applications of such biotechnology approaches, thus further favoring chemical-based interventions including novel insecticides.

**Key Learning Points**

- Indoor residual spraying (IRS) and insecticide-treated bed nets (ITNs) are the most common interventions implemented against sand fly vectors.
- Bioassay records of insecticide resistance (IR) mainly focus on *P*. *argentipes* and *P*. *papatasi* populations from India, the Mediterranean basin, and the Middle East, while there are also sporadic reports for *Lutzomyia* populations in South America.
- Target-site pyrethroid resistance mutations (knockdown resistance (*kdr*) L1014F/S) have been detected in several *Phlebotomus* populations worldwide, while metabolic resistance has not been adequately studied.
- The impact of sand fly IR on control failure needs to be further evaluated to assess risk thresholds for decision-making. Evidence for IR management (IRM) is lacking behind, due to the sporadic IR studies with non-standardized bioassays and the lack of molecular markers for resistance monitoring.
- Several alternative sand fly vector control strategies are in the development pipeline and under evaluation, including attractive toxic sugar baits (ATSBs), repellents, and zooprophylaxis approaches, as well as biotechnology-based approaches in the longer term, but lack evidence with regard to when and where such strategies and/or products could offer greatest public health value. Such alternative strategies would be introduced using an approach of integrated vector management (IVM), in combination with existing products.

**Top five papers**

1. Dhiman RC, Yadav RS. Insecticide resistance in phlebotomine sandflies in Southeast Asia with emphasis on the Indian subcontinent. Infect Dis Poverty. 2016;5(1):106. doi: 10.1186/s40249-016-0200-3. PMID: 27817749; PMCID: PMC5098277.
2. Fotakis EA, Giantsis IA, Demir S, Vontas JG, Chaskopoulou A. Detection of pyrethroid resistance mutations in the major leishmaniasis vector *Phlebotomus papatasi*. J Med Entomol. 2018;55(5):1225–1230. doi: 10.1093/jme/tjy066. PMID: 29912381.
3. Gomes B, Purkait B, Deb RM, Rama A, Singh RP, Foster GM, Coleman M, Kumar V, Paine M, Das P, Weetman D. Knockdown resistance mutations predict DDT resistance and pyrethroid tolerance in the visceral leishmaniasis vector *Phlebotomus argentipes*. PLoS Negl Trop Dis. 2017 Apr 17;11(4):e0005504. doi: 10.1371/journal.pntd.0005504. PMID: 28414744.
4. Hassan MM, Widaa SO, Osman OM, Numiary MS, Ibrahim MA, Abushama HM. Insecticide resistance in the sand fly, *Phlebotomus papatasi* from Khartoum State, Sudan. Parasit Vectors. 2012;5:46. doi: 10.1186/1756-3305-5-46. PMID: 22397726.
5. Huda MM, Ghosh D, Alim A, Almahmud M, Olliaro PL, Matlashewski G, Kroeger A, Mondal D. Intervention packages for early visceral leishmaniasis case detection and sand fly control in Bangladesh: A comparative analysis. Am J Trop Med Hyg. 2019;100(1):97–107. doi: 10.4269/ajtmh.18-0290. PMID: 30457088.

## Supporting information

S1 Table Insecticide resistance bioassay records in *Phlebotomus* and *Lutzomyia* vector species populations worldwide. (XLSX)

S2 Table Reports of *kdr* mutation L1014F/S in *Phlebotomus* spp. populations globally. (XLSX)

## References

[1] Maroli M, Feliciangeli MD, Bichaud L, Charrel RN, Gradoni L. Phlebotomine sandflies and the spreading of leishmaniases and other diseases of public health concern. Med Vet Entomol. 2013;27(2):123–47. doi: 10.1111/j.1365-2915.2012.01034.x . 22924419

[2] Alvar J, Vélez ID, Bern C, Herrero M, Desjeux P, Cano J, et al. WHO Leishmaniasis Control Team. Leishmaniasis worldwide and global estimates of its incidence. PLoS ONE. 2012;7(5): e35671 doi: 10.1371/journal.pone.0035671 22693548 PMC3365071

[3] World Health Organization. Leishmaniasis. 2020. Available from: https://www.who.int/health-topics/leishmaniasis#tab=tab_1.

[4] Oryan A, Akbari M. Worldwide risk factors in leishmaniasis. Asian Pac J Trop Med. 2016;9(10): 925–932. doi: 10.1016/j.apjtm.2016.06.021 . 27794384

[5] Hassaballa IB, Sole CL, Cheseto X, Torto B, Tchouassi DP. Afrotropical sand fly-host plant relationships in a leishmaniasis endemic area, Kenya. PLoS Negl Trop Dis. 2021;15(2):e0009041. doi: 10.1371/journal.pntd.0009041. 33556068 PMC7895382

[6] Malaria Consortium. Leishmaniasis control in eastern Africa: Past and present efforts and future needs. Situation and gap analysis. November 2010. Available from: https://www.malariaconsortium.org/userfiles/file/NTD%20Resources/VL%20EA%20Situation%20Analysis%20Fina_Janl.pdf.

[7] Cecílio P, Oliveira F, da Silva AC. Vaccines for Human Leishmaniasis: Where do we stand and what is still missing? Leishmaniases as re-emerging diseases, Farhat Afrin and Hassan Hemeg, IntechOpen; 2018. Available from: https://www.intechopen.com/books/leishmaniases-as-re-emerging-diseases/vaccines-for-human-leishmaniasis-where-do-we-stand-and-what-is-still-missing-.

[8] Wilson AL, Courtenay O, Kelly-Hope LA, Scott TW, Takken W, Torr SJ, et al. The importance of vector control for the control and elimination of vector-borne diseases. PLoS Negl Trop Dis. 2020;14(1):e0007831. doi: 10.1371/journal.pntd.0007831. 31945061 PMC6964823

[9] Das ML, Roy L, Rijal S, Paudel IS, Picado A, Kroeger A, et al. Comparative study of kala-azar vector control measures in eastern Nepal. Acta Trop. 2010;113(2):162–6. doi: 10.1016/j.actatropica.2009.10.012 . 19879851

[10] Alexander B, Maroli M. Control of phlebotomine sandflies. Med Vet Entomol. 2003;17(1):1–18. doi: 10.1046/j.1365-2915.2003.00420.x . 12680919

[11] Dhiman RC, Yadav RS. Insecticide resistance in phlebotomine sandflies in Southeast Asia with emphasis on the Indian subcontinent. Infect Dis Poverty. 2016;5(1):106. doi: 10.1186/s40249-016-0200-3. 27817749 PMC5098277

[12] Rocha DA, Costa LMD, Pessoa GDC, Obara MT. Methods for detecting insecticide resistance in sand flies: A systematic review. Acta Trop. 2020;213:105747. doi: 10.1016/j.actatropica.2020.105747. 33188748

[13] Huda MM, Ghosh D, Alim A, Almahmud M, Olliaro PL, Matlashewski G, et al. Intervention packages for early visceral leishmaniasis case detection and sandfly control in Bangladesh: A comparative analysis. Am J Trop Med Hyg. 2019;100(1):97–107. doi: 10.4269/ajtmh.18-0290 . 30457088 PMC6335927

[14] Chowdhury R, Huda MM, Kumar V, Das P, Joshi AB, Banjara MR, et al. The Indian and Nepalese programmes of indoor residual spraying for the elimination of visceral leishmaniasis: performance and effectiveness. Ann Trop Med Parasitol. 2011;105(1):31–5. doi: 10.1179/136485911X12899838683124 . 21294947 PMC4089790

[15] Faraj C, Adlaoui el B, Ouahabi S, Elkohli M, Elrhazi M, Laqraa L, et al. Field evaluation of alpha-cypermethrin in indoor residual spraying for leishmaniasis control in an endemic area, northern Morocco. Parasit Vectors. 2013;6:354. doi: 10.1186/1756-3305-6-354. 24330760 PMC4029413

[16] Yaghoobi-Ershadi MR. Control of Phlebotomine Sand Flies in Iran: A Review Article. J Arthropod Borne Dis. 2016;10(4):429–44. . 28032095 PMC5186733

[17] World Health Organization. Global insecticide use for vector-borne disease control: a 10-year assessment [2000–2009], 5th ed. 2011. Available from: https://apps.who.int/iris/handle/10665/44670.

[18] Joshi AB, Das ML, Akhter S, Chowdhury R, Mondal D, Kumar V, et al. Chemical and environmental vector control as a contribution to the elimination of visceral leishmaniasis on the Indian subcontinent: cluster randomized controlled trials in Bangladesh. India and Nepal BMC Med. 2009;7:54. doi: 10.1186/1741-7015-7-54. 19804620 PMC2763005

[19] Chowdhury R, Faria S, Huda MM, Chowdhury V, Maheswary NP, Mondal D, et al. Control of *Phlebotomus argentipes* (Diptera: Psychodidae) sand fly in Bangladesh: A cluster randomized controlled trial. PLoS Negl Trop Dis. 2017;11(9):e0005890. doi: 10.1371/journal.pntd.0005890. 28873425 PMC5600390

[20] Banjara MR, Das ML, Gurung CK, Singh VK, Joshi AB, Matlashewski G, et al. Integrating case detection of visceral leishmaniasis and other febrile illness with vector control in the post-elimination phase in Nepal. Am J Trop Med Hyg. 2019;100(1):108–14. doi: 10.4269/ajtmh.18-0307 . 30426921 PMC6335889

[21] Feliciangeli MD, Mazzarri MB, Campbell-Lendrum D, Maroli M, Maingon R. Cutaneous leishmaniasis vector control perspectives using lambda-cyhalothrin residual house spraying in El Ingenio, Miranda State, Venezuela. Trans R Soc Trop Med Hyg. 2003;97(6):641–6. doi: 10.1016/s0035-9203(03)80095-0 . 16117955

[22] Feliciangeli MD, Mazzarri MB, Blas SS, Zerpa O. Control trial of *Lutzomyia longipalpis* s.l. in the Island of Margarita, Venezuela. Tropical Med Int Health. 2003;8(12):1131–6. doi: 10.1046/j.1360-2276.2003.01137.x . 14641849

[23] Messenger LA, Rowland M. Insecticide-treated durable wall lining (ITWL): future prospects for control of malaria and other vector-borne diseases. Malar J. 2017;16(1):213. doi: 10.1186/s12936-017-1867-z. 28532494 PMC5441104

[24] Mondal D, Das ML, Kumar V, Huda MM, Das P, Ghosh D, et al. Efficacy, safety and cost of insecticide treated wall lining, insecticide treated bed nets and indoor wall wash with lime for visceral leishmaniasis vector control in the Indian Sub-continent: A multi-country cluster randomized controlled trial. PLoS Negl Trop Dis. 2016;10(8):e0004932. doi: 10.1371/journal.pntd.0004932. 27533097 PMC4988640

[25] Huda MM, Kumar V, Das ML, Ghosh D, Priyanka J, Das P, et al. Entomological efficacy of durable wall lining with reduced wall surface coverage for strengthening visceral leishmaniasis vector control in Bangladesh. India and Nepal BMC Infect Dis. 2016;16(1):539. doi: 10.1186/s12879-016-1881-8. 27716091 PMC5052807

[26] Samuel M, Maoz D, Manrique P, Ward T, Runge-Ranzinger S, Toledo J, et al. Community effectiveness of indoor spraying as a dengue vector control method: A systematic review. PLoS Negl Trop Dis. 2017;11(8):e0005837. doi: 10.1371/journal.pntd.0005837. 28859087 PMC5578493

[27] Ostyn B, Vanlerberghe V, Picado A, Dinesh DS, Sundar S, Chappuis F, et al. Vector control by insecticide-treated nets in the fight against visceral leishmaniasis in the Indian subcontinent, what is the evidence? Tropical Med Int Health. 2008;13(8):1073–85. doi: 10.1111/j.1365-3156.2008.02110.x . 18564350

[28] Jalouk L, Al Ahmed M, Gradoni L, Maroli M. Insecticide-treated bednets to prevent anthroponotic cutaneous leishmaniasis in Aleppo Governorate, Syria: results from two trials. Trans R Soc Trop Med Hyg. 2007;101(4):360–7. doi: 10.1016/j.trstmh.2006.07.011 . 17097698

[29] Ritmeijer K, Davies C, van Zorge R, Wang SJ, Schorscher J, Dongu’du SI, et al. Evaluation of a mass distribution programme for fine-mesh impregnated bednets against visceral leishmaniasis in eastern Sudan. Tropical Med Int Health. 2007;12(3):404–14. doi: 10.1111/j.1365-3156.2006.01807.x . 17313512

[30] Emami MM, Yazdi M, Guillet P. Efficacy of Olyset long-lasting bednets to control transmission of cutaneous leishmaniasis in Iran. East Mediterr Health J. 2009;15(5):1075–83. . 20214120

[31] Moosa-Kazemi SH, Yaghoobi-Ershadir MR, Akhavan AA, Abdoli H, Zahraei-Ramazani AR, Jafari R, et al. Deltamethrin-impregnated bed nets and curtains in an anthroponotic cutaneous leishmaniasis control program in northeastern Iran. Ann Saudi Med. 2007;27(1): 6–12. doi: 10.5144/0256-4947.2007.6 17277492 PMC6077027

[32] Yaghoobi-Ershadi MR, Moosa-Kazemi SH, Zahraei-Ramazani AR, Jalai-Zand AR, Akhavan AA, Arandain MH, et al. Evaluation of deltamethrin-impregnated bed nets and curtains for control of zoonotic cutaneous leishmaniasis in a hyperendemic area of Iran. Bull Soc Pathol Exot. 2006;99(1):43–8. doi: 10.3185/pathexo2818 . 16568684

[33] Alten B, Caglar SS, Kaynas S, Simsek FM. Evaluation of protective efficacy of K-OTAB impregnated bednets for cutaneous leishmaniasis control in Southeast Anatolia-Turkey. J Vector Ecol. 2003;28(1):53–64. . 12831129

[34] Mondal D, Chowdhury R, Huda MM, Maheswary NP, Akther S, Petzold M, et al. Insecticide-treated bed nets in rural Bangladesh: their potential role in the visceral leishmaniasis elimination programme. Tropical Med Int Health. 2010;15(11):1382–9. doi: 10.1111/j.1365-3156.2010.02635.x . 20946233

[35] Chowdhury R, Chowdhury V, Faria S, Akter S, Dash AP, Bhattacharya SK, et al. Effect of insecticide-treated bed nets on visceral leishmaniasis incidence in Bangladesh. A retrospective cohort analysis. PLoS Negl Trop Dis. 2019;13(9): e0007724. doi: 10.1371/journal.pntd.0007724 31525195 PMC6762203

[36] Picado A, Dash AP, Bhattacharya S, Boelaert M. Vector control interventions for visceral leishmaniasis elimination initiative in South Asia, 2005–2010. Indian J Med Res. 2012;136(1):22–31. . 22885260 PMC3461713

[37] Gunay F, Karakus M, Oguz G, Dogan M, Karakaya Y, Ergan G, et al. Evaluation of the efficacy of Olyset Plus in a village-based cohort study in the Cukurova Plain, Turkey, in an area of hyper-endemic cutaneous leishmaniasis. J Vector Ecol. 2014;39(2):395–405. doi: 10.1111/jvec.12115 . 25424269

[38] Picado A, Das ML, Kumar V, Kesari S, Dinesh DS, Roy L, et al. Effect of village-wide use of long-lasting insecticidal nets on visceral leishmaniasis vectors in India and Nepal: a cluster randomized trial. PLoS Negl Trop Dis. 2010;4(1):e587. doi: 10.1371/journal.pntd.0000587. 20126269 PMC2811172

[39] Chowdhury R, Kumar V, Mondal D, Das ML, Das P, Dash AP, et al. Implication of vector characteristics of *Phlebotomus argentipes* in the kala-azar elimination programme in the Indian sub-continent. Pathog Glob Health. 2016;110(3):87–96. doi: 10.1080/20477724.2016.1180775 . 27376500 PMC4940889

[40] Picado A, Ostyn B, Rijal S, Sundar S, Singh SP, Chappuis F, et al. Long-lasting insecticidal nets to prevent visceral leishmaniasis in the Indian subcontinent; Methodological lessons learned from a cluster randomised controlled trial. PLoS Negl Trop Dis. 2015;9(4):e0003597. doi: 10.1371/journal.pntd.0003597. 25856238 PMC4391877

[41] Banks SD, Murray N, Wilder-Smith A, Logan JG. Insecticide-treated clothes for the control of vector-borne diseases: a review on effectiveness and safety. Med Vet Entomol. 2014;28(Suppl 1):14–25. doi: 10.1111/mve.12068 . 24912919

[42] Faiman R, Cuño R, Warburg A. Control of phlebotomine sand flies with vertical fine-mesh nets. J Med Entomol. 2009;46(4):820–31. doi: 10.1603/033.046.0412 . 19645284

[43] Faiman R, Kirstein O, Freund M, Guetta H, Warburg A. Exclusion of phlebotomine sand flies from inhabited areas by means of vertical mesh barriers. Trans R Soc Trop Med Hyg. 2011;105(9):512–8. doi: 10.1016/j.trstmh.2011.05.011 . 21752415

[44] Britch SC, Linthicum KJ, Walker TW, Farooq M, Gordon SW, Clark JW, et al. Evaluation of ULV applications against Old World sand fly (Diptera: Psychodidae) species in equatorial Kenya. J Med Entomol. 2011;48(6):1145–59. doi: 10.1603/me11025 . 22238873

[45] Chaskopoulou A, Miaoulis M, Kashefi J. Ground ultra low volume (ULV) space spray applications for the control of wild sand fly populations (Psychodidae: Phlebotominae) in Europe. Acta Trop. 2018;182:54–9. doi: 10.1016/j.actatropica.2018.02.003 . 29457992

[46] Qualls WA, Müller GC, Khallaayoune K, Revay EE, Zhioua E, Kravchenko VD, et al. Control of sand flies with attractive toxic sugar baits (ATSB) and potential impact on non-target organisms in Morocco. Parasit Vectors. 2015;8:87. doi: 10.1186/s13071-015-0671-2. 25890039 PMC4333173

[47] Schlein Y, Müller GC. Experimental control of *Phlebotomus papatasi* by spraying attractive toxic sugar bait (ATSB) on vegetation. Trans R Soc Trop Med Hyg. 2010;104(12):766–71. doi: 10.1016/j.trstmh.2010.08.014 . 20889177

[48] Müller GC, Schlein Y. Different methods of using attractive sugar baits (ATSB) for the control of *Phlebotomus papatasi*. J Vector Ecol. 2011;36(Suppl 1):S64–70. doi: 10.1111/j.1948-7134.2011.00113.x . 21366782

[49] Saghafipour A, Vatandoost H, Zahraei-Ramazani AR, Yaghoobi-Ershadi MR, Rassi Y, Karami Jooshin M, et al. Control of zoonotic cutaneous leishmaniasis vector, *Phlebotomus papatasi*, using attractive toxic sugar baits (ATSB). PLoS ONE. 2017;12(4):e0173558. doi: 10.1371/journal.pone.0173558. 28426679 PMC5398489

[50] Saghafipour A, Vatandoost H, Zahraei-Ramazani AR, Yaghoobi-Ershadi MR, Rassi Y, Shirzadi MR, et al. Bioassay evaluation of residual activity of attractive toxic sugar-treated barrier fence in the control of *Phlebotomus papatasi* (Diptera: Psychodidae). J Vector Borne Dis. 2016;53(4):335–40. . 28035110

[51] Weeks ENI, Wasserberg G, Logan JL, Agneessens J, Stewart SA, Dewhirst S. Efficacy of the insect repellent IR3535 on the sand fly *Phlebotomus papatasi* in human volunteers. J Vector Ecol. 2019;44(2):290–2. doi: 10.1111/jvec.12362 . 31729794

[52] Dinesh DS, Kumari S, Kumar V, Das P. The potentiality of botanicals and their products as an alternative to chemical insecticides to sandflies (Diptera: Psychodidae): a review. J Vector Borne Dis. 2014;51(1):1–7. . 24717195

[53] Giantsis IA, Chaskopoulou A. Broadening the tools for studying sand fly breeding habitats: A novel molecular approach for the detection of phlebotomine larval DNA in soil substrates. Acta Trop. 2019;190:123–8. doi: 10.1016/j.actatropica.2018.11.008 . 30444972

[54] Gómez-Bravo A, Alvarez-Costa A, Fronza G, Abril M, Zerba EN, Juan LW. High effectiveness of an adulticide-larvicide formulation for field control of sandflies (Diptera: Psychodidae) in the city of Clorinda. Argentina Parasite Epidemiol Control. 2019;7:e00110. doi: 10.1016/j.parepi.2019.e00110. 31236488 PMC6581875

[55] Ngure PK, Kasili S, Anjili CO, Karanja RM, Kaburi J, Mwangi M, et al. Effects of *Metarhizium anisopliae* on sand fly populations in their natural habitats in Marigat sub- County, Baringo County. Kenya Afr J Health Sci. 2015;29:398–407.

[56] Mascari TM, Mitchell MA, Rowton ED, Foil LD. Evaluation of juvenile hormone analogues as rodent feed-through insecticides for control of immature phlebotomine sandflies. Med Vet Entomol. 2011;25(2):227–31. doi: 10.1111/j.1365-2915.2010.00919.x . 21073493

[57] Hanafi HA, Szumlas DE, Fryauff DJ, El-Hossary SS, Singer GA, Osman SG, et al. Effects of ivermectin on blood-feeding *Phlebotomus papatasi*, and the promastigote stage of Leishmania major. Vector Borne Zoonotic Dis. 2011;11(1):43–52. doi: 10.1089/vbz.2009.0030 . 20518644

[58] Mekuriaw W, Balkew M, Messenger LA, Yewhalaw D, Woyessa A, Massebo F. The effect of ivermectin on fertility, fecundity and mortality of *Anopheles arabiensis* fed on treated men in Ethiopia. Malar J. 2019;18(1):357. doi: 10.1186/s12936-019-2988-3. 31703736 PMC6842263

[59] Mascari TM, Mitchell MA, Rowton ED, Foil LD. Ivermectin as a rodent feed-through insecticide for control of immature sand flies (Diptera: Psychodidae). J Am Mosq Control Assoc. 2008;24(2):323–6. doi: 10.2987/5678.1 . 18666544

[60] Poché RM, Burruss D, Polyakova L, et al. Treatment of livestock with systemic insecticides for control of *Anopheles arabiensis* in western Kenya. Malar J. 2015;14:351. doi: 10.1186/s12936-015-0883-0. 26377691 PMC4574316

[61] Ingenloff K, Garlapati R, Poché D, Singh MI, Remmers JL, Poché RM. Feed-through insecticides for the control of the sand fly Phlebotomus argentipes. Med Vet Entomol. 2013;27(1):10–8. doi: 10.1111/j.1365-2915.2012.00995.x . 23278322

[62] Poché RM, Garlapati R, Singh MI, Poché DM. Evaluation of fipronil oral dosing to cattle for control of adult and larval sand flies under controlled conditions. J Med Entomol. 2013;50(4):833–7. . 23926782

[63] Derbali M, Polyakova L, Boujaâma A, Burruss D, Cherni S, Barhoumi W, et al. Laboratory and field evaluation of rodent bait treated with fipronil for feed through and systemic control of *Phlebotomus papatasi*. Acta Trop. 2014;135:27–32. doi: 10.1016/j.actatropica.2014.03.013 . 24681222

[64] Poché DM, Wang H-H, Grant WE. Visceral leishmaniasis on the Indian Subcontinent: Efficacy of fipronil-based cattle treatment in controlling sand fly populations is dependent on specific aspects of sand fly ecology. PLoS Negl Trop Dis. 2020;14(2):e0008011. doi: 10.1371/journal.pntd.0008011. 32069283 PMC7048295

[65] Gálvez R, Montoya A, Fontal F. Martínez De Murguía L, Miró G. Controlling phlebotomine sand flies to prevent canine *Leishmania infantum* infection: A case of knowing your enemy. Res Vet Sci. 2018;121:94–103. doi: 10.1016/j.rvsc.2018.10.008 . 30366124

[66] Yimam Y, Mohebali M. Effectiveness of insecticide-impregnated dog collars in reducing incidence rate of canine visceral leishmaniasis: A systematic review and meta-analysis. PLoS ONE. 2020;15(9):e0238601. doi: 10.1371/journal.pone.0238601. 32881961 PMC7470253

[67] Courtenay O, Bazmani A, Parvizi P, Ready PD, Cameron MM. Insecticide-impregnated dog collars reduce infantile clinical visceral leishmaniasis under operational conditions in NW Iran: A community-wide cluster randomised trial. PLoS Negl Trop Dis. 2019;13(3):e0007193. doi: 10.1371/journal.pntd.0007193. 30830929 PMC6417739

[68] Reguera RM, Morán M, Pérez-Pertejo Y, García-Estrada C, Balaña-Fouce R. Current status on prevention and treatment of canine leishmaniasis. Vet Parasitol. 2016;227:98–114. doi: 10.1016/j.vetpar.2016.07.011 . 27523945

[69] Li AY, Perez de Leon AA, Linthicum KJ, Britch SC, Bast JD, Debboun M. Baseline susceptibility to pyrethroid and organophosphate insecticides in two Old World sand fly species (Diptera: Psychodidae). US Army Med Dep J. 2015:3–9. . 26276940

[70] WHO, 2016. Test procedures for insecticide resistance monitoring in malaria vector mosquitoes, 2nd ed. World Health Organization, Geneva, Switzerland. Available from: http://www.who.int/malaria/areas/vector_control/insecticide_resistance/en/.

[71] Brogdon WG, Chan A. Guidelines for evaluating insecticide resistance in vectors using the CDC bottle bioassay. CDC technical report. Methods in *Anopheles* research, 2nd ed. Atlanta: Centers for Disease Control and Prevention; 2010.

[72] Denlinger DS, Lozano-Fuentes S, Lawyer PG, Black WC 4th, Bernhardt SA. Assessing insecticide susceptibility of laboratory *Lutzomyia longipalpis* and *Phlebotomus papatasi* sand flies (Diptera: Psychodidae: Phlebotominae). J Med Entomol. 2015;52(5):1003–12. doi: 10.1093/jme/tjv091 . 26336231 PMC4574604

[73] Denlinger DS, Creswell JA, Anderson JL, Reese CK, Bernhardt SA. Diagnostic doses and times for *Phlebotomus papatasi* and *Lutzomyia longipalpis* sand flies (Diptera: Psychodidae: Phlebotominae) using the CDC bottle bioassay to assess insecticide resistance. Parasit Vectors. 2016;9:212. doi: 10.1186/s13071-016-1496-3. 27083417 PMC4833940

[74] González MA, Bell MJ, Bernhardt SA, Brazil RP, Dilger E, Courtenay O, et al. Susceptibility of wild-caught *Lutzomyia longipalpis* (Diptera: Psychodidae) sand flies to insecticide after an extended period of exposure in western São Paulo. Brazil Parasit Vectors. 2019;12(1):110. doi: 10.1186/s13071-019-3364-4. 30871639 PMC6419423

[75] Gomes B, Purkait B, Deb RM, Rama A, Singh RP, Foster GM, et al. Knockdown resistance mutations predict DDT resistance and pyrethroid tolerance in the visceral leishmaniasis vector *Phlebotomus argentipes*. PLoS Negl Trop Dis. 2017;11(4):e0005504. doi: 10.1371/journal.pntd.0005504. 28414744 PMC5407848

[76] Sardar AA, Saha P, Chatterjee M, Bera DK, Biswas P, Maji D, et al. Insecticide susceptibility status of *Phlebotomus argentipes* and polymorphisms in voltage-gated sodium channel (vgsc) gene in kala-azar endemic areas of West Bengal. India Acta Trop. 2018;185:285–93. doi: 10.1016/j.actatropica.2018.06.005 . 29890155

[77] Mandal R, Kumar V, Kesari S, Das P. Assessing the combined effects of household type and insecticide effectiveness for kala-azar vector control using indoor residual spraying: a case study from North Bihar. India Parasit Vectors. 2019;12(1):409. doi: 10.1186/s13071-019-3670-x. 31439002 PMC6705094

[78] Chowdhury R, Das ML, Chowdhury V, Roy L, Faria S, Priyanka J, et al. Susceptibility of field-collected *Phlebotomus argentipes* (Diptera: Psychodidae) sand flies from Bangladesh and Nepal to different insecticides. Parasit Vectors. 2018;11(1):336. doi: 10.1186/s13071-018-2913-6. 29866195 PMC5987452

[79] Pathirage DRK, Karunaratne SHPP, Senanayake SC, Karunaweera ND. Insecticide susceptibility of the sand fly leishmaniasis vector *Phlebotomus argentipes* in Sri Lanka. Parasit Vectors. 2020;13(1):246. doi: 10.1186/s13071-020-04117-y. 32404115 PMC7218544

[80] Maroli M, Cianchi T, Bianchi R, Khoury C. Testing insecticide susceptibility of *Phlebotomus perniciosus* and *P. papatasi* (Diptera: Psychodidae) in Italy. Ann Ist Super Sanita. 2002;38(4):419–23. . 12760339

[81] Karakus M, Gocmen B, Özbel Y. Insecticide susceptibility status of wild-caught sand fly populations collected from two leishmaniasis endemic areas in western Turkey. J Arthropod Borne Dis. 2017;11(1):86–94. . 29026855 PMC5629309

[82] Karakuş M, Sarıkaya Y, Oğuz G, Doğan M, Ergan G, Günay F, et al. Assessment of diagnostic doses for widely used synthetic pyrethroids (Deltamethrin & Permethrin) in an endemic focus of leishmaniasis in Turkey. Parasit Vectors. 2016;9(1):526. doi: 10.1186/s13071-016-1812-y. 27688146 PMC5043626

[83] Faraj C, Ouahabi S, Adlaoui el B, El Elkohli M, Lakraa L, El Rhazi M, et al. Insecticide susceptibility status of *Phlebotomus (Paraphlebotomus) sergenti* and *Phlebotomus (Phlebotomus) papatasi* in endemic foci of cutaneous leishmaniasis in Morocco. Parasit Vectors 2012;5: 51. doi: 10.1186/1756-3305-5-51. 22429776 PMC3359231

[84] World Health Organization. Instructions for determining the susceptibility or resistance of adult blackflies, sandflies and biting midges to insecticides: WHO/VBC/81.810. 1981.

[85] Hassan MM, Widaa SO, Osman OM, Numiary MS, Ibrahim MA, Abushama HM. Insecticide resistance in the sand fly, Phlebotomus papatasi from Khartoum State. Sudan Parasit Vectors. 2012;5:46. doi: 10.1186/1756-3305-5-46. 22397726 PMC3314797

[86] Afshar AA, Rassi Y, Sharifi I, Abai M, Oshaghi M, Yaghoobi-Ershadi M, et al. Susceptibility status of *Phlebotomus papatasi* and *P. sergenti* (Diptera: Psychodidae) to DDT and deltamethrin in a focus of cutaneous leishmaniasis after earthquake strike in Bam, Iran. Iran J Arthropod Borne Dis. 2011;5(2):32–41. . 22808416 PMC3385580

[87] Saeidi Z, Vatandoost H, Akhavan AA, Yaghoobi-Ershadi MR, Rassi Y, Sheikh Z, et al. Baseline susceptibility of a wild strain of Phlebotomus papatasi (Diptera: Psychodidae) to DDT and pyrethroids in an endemic focus of zoonotic cutaneous leishmaniasis in Iran. Pest Manag Sci. 2012;68(5):669–75. doi: 10.1002/ps.2278 . 22351603

[88] Saeidi Z, Vatandoost H, Akhavan AA, Yaghoobi-Ershadi MR, Rassi Y, Arandian MH, et al. Baseline insecticide susceptibility data of *Phlebotomus papatasi* in Iran. J Vector Borne Dis. 2013 Mar;50(1):57–61. . 23703441

[89] Shirani-Bidabadi L, Zahraei-Ramazani A, Yaghoobi-Ershadi MR, Rassi Y, Akhavan AA, Oshaghi MA, et al. Assessing the insecticide susceptibility status of field population of *Phlebotomus papatasi* (Diptera: Psychodidae) in a hyperendemic area of zoonotic cutaneous leishmaniasis in Esfahan Province. Central Iran Acta Trop. 2017;176:316–22. doi: 10.1016/j.actatropica.2017.08.035 . 28870534

[90] Arzamani K, Vatandoost H, Rassi Y, Abai MR, Akhavan AA, Alavinia M, et al. Susceptibility status of wild population of *Phlebotomus sergenti* (Diptera: Psychodidae) to different imagicides in an endemic focus of cutaneous leishmaniasis in northeast of Iran. J Vector Borne Dis. 2017;54(3):282–6. doi: 10.4103/0972-9062.217621 . 29097645

[91] Alexander B, Barros VC, de Souza SF, Barros SS, Teodoro LP, Soares ZR, et al. Susceptibility to chemical insecticides of two Brazilian populations of the visceral leishmaniasis vector *Lutzomyia longipalpis* (Diptera: Psychodidae). Tropical Med Int Health. 2009;14(10):1272–7. doi: 10.1111/j.1365-3156.2009.02371.x . 19772549

[92] Rocha DA, Andrade AJ, Moura LR, Figueiredo NG, Pessoa GCD, Obara MT. Susceptibility of phlebotomine sandflies (Diptera: Psychodidae) collected in the field, to alpha-cypermethrin in four municipalities endemic to leishmaniasis. Rev Inst Med Trop Sao Paulo. 2020;62:e38. doi: 10.1590/S1678-9946202062038. 32520213 PMC7274764

[93] Pessoa GC, Lopes JV, Rocha MF, Pinheiro LC, Rosa AC, Michalsky ÉM, et al. Baseline susceptibility to alpha-cypermethrin in *Lutzomyia longipalpis* (Lutz & Neiva, 1912) from Lapinha Cave (Brazil). Parasit Vectors. 2015;8:469. doi: 10.1186/s13071-015-1076-y. 26381242 PMC4573933

[94] Álvarez L, Duran Y, González A, Suárez J, Oviedo M. Concentraciones letales (CL_50_ y CL_95_) y dosis diagnósticas de fenitrotion y lambdacialotrina para *Lutzomyia evansi* (Diptera: Psychodidae) de los Pajones, estado Trujillo. Venezuela Bol Mal Salud Amb. 2006;46(1):31–7. ISSN: 1690-4648.

[95] Henriquez C, Pereira Y, Cochero S, Bejarano EE. Dosis diagnóstica y umbral de resistencia de *Lutzomyia evansi* (Diptera: Psychodidae), a dos insecticidas utilizados en salud pública en Colombia: deltametrina y lambdacihalotrina. Rev Soc Entomol Argent. 2009;68(3–4):287–94. ISSN: 0373-5680.

[96] Vargas VF. Franklin, Córdova PO, Alvarado AA. Determinación de la resistencia a insecticidas en Aedes aegypti, Anopheles albimanus y Lutzomyia peruensis procedentes del Norte Peruano. Rev perú med exp salud publica. 2006;23(4):259–64. ISSN: 1726-4634.

[97] Balabanidou V, Kefi M, Aivaliotis M, Koidou V, Girotti JR, Mijailovsky SJ, et al. Mosquitoes cloak their legs to resist insecticides. Proc Biol Sci. 2019;286(1907):20191091. doi: 10.1098/rspb.2019.1091. 31311476 PMC6661348

[98] Carrasco D, Lefèvre T, Moiroux N, Pennetier C, Chandre F, Cohuet A. Behavioural adaptations of mosquito vectors to insecticide control. Curr Opin Insect Sci. 2019; 34:48–54. doi: 10.1016/j.cois.2019.03.005 . 31247417

[99] Liu N. Insecticide resistance in mosquitoes: impact, mechanisms, and research directions. Annu Rev Entomol. 2015;60:537–59. doi: 10.1146/annurev-ento-010814-020828 . 25564745

[100] Du Y, Nomura Y, Satar G, Hu Z, Nauen R, He SY, et al. Molecular evidence for dual pyrethroid-receptor sites on a mosquito sodium channel. Proc Natl Acad Sci U S A. 2013;110(29):11785–90. doi: 10.1073/pnas.1305118110 . 23821746 PMC3718148

[101] Fotakis EA, Giantsis IA, Demir S, Vontas JG, Chaskopoulou A. Detection of pyrethroid resistance mutations in the major leishmaniasis vector *Phlebotomus papatasi*. J Med Entomol. 2018;55(5):1225–30. doi: 10.1093/jme/tjy066 . 29912381

[102] Fotakis EA, Giantsis IA, Castells Sierra J, Tanti F, Balaska S, Mavridis K, et al. Population dynamics, pathogen detection and insecticide resistance of mosquito and sand fly in refugee camps. Greece Infect Dis Poverty. 2020;9(1):30. doi: 10.1186/s40249-020-0635-4. 32183909 PMC7079361

[103] Surendran SN, Karunaratne SH, Adams Z, Hemingway J, Hawkes NJ. Molecular and biochemical characterization of a sand fly population from Sri Lanka: evidence for insecticide resistance due to altered esterases and insensitive acetylcholinesterase. Bull Entomol Res. 2005;95(4):371–80. doi: 10.1079/ber2005368 . 16048685

[104] Moyes CL, Vontas J, Martins AJ, Ng LC, Koou SY, Dusfour I, et al. Contemporary status of insecticide resistance in the major Aedes vectors of arboviruses infecting humans. PLoS Negl Trop Dis. 2017;11(7):e0005625. doi: 10.1371/journal.pntd.0005625. 28727779 PMC5518996

[105] Coutinho-Abreu IV, Balbino VQ, Valenzuela JG, Sonoda IV, Ramalho-Ortigão JM. Structural characterization of acetylcholinesterase 1 from the sand fly *Lutzomyia longipalpis* (Diptera: Psychodidae). J Med Entomol. 2007;44(4):639–50. doi: 10.1603/0022-2585(2007)44[639:scoaft]2.0.co;2 . 17695019

[106] Temeyer KB, Brake DK, Tuckow AP, Li AY. Pérez de León AA. Acetylcholinesterase of the sand fly, *Phlebotomus papatasi* (Scopoli): cDNA sequence, baculovirus expression, and biochemical properties. Parasit Vectors. 2013;6:31. doi: 10.1186/1756-3305-6-31. 23379291 PMC3598880

[107] Vontas J, Katsavou E, Mavridis K. Cytochrome P450-based metabolic insecticide resistance in Anopheles and Aedes mosquito vectors: Muddying the waters. Pestic Biochem Physiol. 2020;170:104666. doi: 10.1016/j.pestbp.2020.104666. 32980073

[108] Samantsidis GR, Panteleri R, Denecke S, Kounadi S, Christou I, Nauen R, et al. ’What I cannot create, I do not understand’: functionally validated synergism of metabolic and target site insecticide resistance. Proc Biol Sci. 2020;287(1927):20200838. doi: 10.1098/rspb.2020.0838. 32453986 PMC7287358

[109] Fawaz EY, Zayed AB, Fahmy NT, Villinski JT, Hoel DF, Diclaro JW 2nd. Pyrethroid insecticide resistance mechanisms in the adult *Phlebotomus papatasi* (Diptera: Psychodidae). J Med Entomol. 2016;53(3):620–8. doi: 10.1093/jme/tjv256 . 26810731

[110] Louradour I, Ghosh K, Inbar E, Sacks DL. CRISPR/Cas9 mutagenesis in *Phlebotomus papatasi*: the Immune deficiency pathway impacts vector competence for *Leishmania major*. mBio. 2019;10(4): e01941–19. doi: 10.1128/mBio.01941-19 . 31455654 PMC6712399

[111] Ono M, Braig HR, Munstermann LE, Ferro C, O’Neill SL. Wolbachia infections of phlebotomine sand flies (Diptera: Psychodidae). J Med Entomol. 2001;38(2):237–41. doi: 10.1603/0022-2585-38.2.237 . 11296829

[112] Zhang D, Lees RS, Xi Z, Gilles JRL, Bourtzis K. Combining the sterile insect technique with Wolbachia-based approaches: II- a safer approach to Aedes albopictus population suppression programmes, designed to minimize the consequences of inadvertent female release. PLoS ONE. 2015;10(8):e0135194. doi: 10.1371/journal.pone.0135194 26252474 PMC4529199

[113] Kelly PH, Bahr SM, Serafim TD, Ajami NJ, Petrosino JF. Meneses C, et al. The gut microbiome of the vector Lutzomyia longipalpis is essential for survival of Leishmania infantum. mBio. 2017;8(1): e01121–16. doi: 10.1128/mBio.01121-16 . 28096483 PMC5241394

